# The difference in light intensities during culture affects the production of health-beneficial metabolites in a diatom used in producing aquaculture feed

**DOI:** 10.1038/s41598-026-37956-3

**Published:** 2026-01-31

**Authors:** Hiroaki Takebe, Atsushi Sakurai, Sousuke Imamura

**Affiliations:** Space Environment and Energy Laboratories, NTT, Inc., Musashino-shi, Tokyo, 180- 8585 Japan

**Keywords:** Microalgae, Aquaculture, *Chaetoceros gracilis*, Metabolomics, Health-beneficial metabolites, Applied microbiology, Plant stress responses, Metabolomics

## Abstract

Microalgae are increasingly being utilized as sustainable materials for aquaculture feed production. As microalgae-derived compounds, including those with health benefits to humans, can accumulate in the bodies of fish and shellfish, enhancing the production of these compounds will further add value to the utilization of microalgae as aquaculture feed. Although light intensity has been known to affect the composition of intracellular compounds, the relationship between light intensity and the production of health-beneficial metabolites in microalgae remains unclear. Hence, in this study, the changes in the production of water- and lipid-soluble compounds in *Chaetoceros gracilis*, a diatom species used as aquaculture feed, under high and normal light conditions were quantified by performing metabolome analyses. While there was no significant difference in the growth of *C. gracilis* between the light conditions, the overall composition of compounds differed between the light intensities, and several health-beneficial metabolites were specifically produced under each light condition. Interestingly, these included compounds such as nobiletin and carnosine, which are not commonly reported to be produced by microalgae. Our results suggest the potential that by varying light intensity, we selectively modulate the types and amounts of health-beneficial metabolites in microalgal cells without altering the overall yield of the feed.

**Supplementary Information:**

The online version contains supplementary material available at 10.1038/s41598-026-37956-3.

## Introduction

As microalgae only require light, water, and carbon dioxide to exhibit autotrophic growth, they are considered sustainable biological materials due to their low environmental impact^1^. For instance, microalgae have been recently utilized in various industries, including aquaculture, fuel, and food production^2–4^; of these industries, microalgae are in high demand in aquaculture. Aquaculture accounts for 49.2% of the global fishery harvest, and its demand continues to increase^5^. However, as traditional feed sources such as fishmeal have a high environmental impact and, thus, are not sustainable^6–9^, microalgae have gained attention as a substitute. Therefore, it is imperative to develop better methods of producing feed made from microalgae.

Aside from yield and efficiency, the chemical composition of the feed is crucial in aquaculture feed production. Some microalgae-derived compounds that have health benefits for both fish and humans accumulate in the bodies of predatory fish. For example, astaxanthin, an industrially important antioxidant that imparts a vibrant pink color to salmon flesh, is derived from microalgae^10^; through the food web, it is incorporated into salmon and later on accumulates in their bodies as a result of bioconcentration^10^. Thus, the compounds present in microalgal feed significantly influence seafood, either indirectly or directly, enhancing the value of both the seafood and the feed itself. Therefore, it is essential to understand the types and quantities of health-beneficial metabolites to human present in the cells of microalgal species used as feed.

Light intensity is among the crucial factors that influence the quantity and variety of compounds in microalgae. Light intensity is closely related to photosynthesis, the mechanism of energy and compound production within microalgal cells^11^. Generally, increasing light intensity enhances growth and, thus, increases the net production of compounds; however, exceeding a certain light intensity causes photoinhibition, resulting in growth stagnation or even cell death^12,13^. Moreover, light intensity also affects the production of specific compounds. For example, it has been reported that light intensity influences the production of lipids such as α-linolenic acid as well as pigments such as astaxanthin and fucoxanthin in microalgal cells^10,14–16^. Various methods have been developed utilizing this mechanism to control the production of specific compound groups, such as lipids and pigments, by controlling light intensity in several microalgal species, including commercial species such as *Euglena* (Subphylum Euglenoida) and *Haematococcus* (Phylum Chlorophyta)^15,17–20^. However, the relationship between cultivation light intensity and the production of health-beneficial metabolites in microalgae used for aquaculture feed remains unknown.

Among the microalgae used for aquaculture feed, the marine diatom *Chaetoceros gracilis* has been recognized as an important species; it has been widely cultured as feed for shrimp larvae and bivalves such as oysters^21–23^. Recently, it has been reported that controlling nutrient supplementation and light intensity can increase the production of fucoxanthin and eicosapentaenoic acid in this species^16^. However, *C. gracilis* has not yet been subjected to comprehensive metabolite analyses, and the changes in the types and amounts of health-beneficial metabolites it produces under varying light intensities remain unclear. Additionally, although it would vary by compound group, low-molecular-weight (LMW) compounds are generally considered to have higher bioavailability^24–26^. Therefore, a comprehensive analysis of health-beneficial LMW compounds should be given particular attention from the perspective of bioavailability in the bodies of predatory fish and ultimately human consumers.

Therefore, this study aimed to determine whether the types and amounts of health-beneficial LMW compounds accumulate in the cells of *C. gracilis* cultured under different light intensities (50 and 250 µmol photon/m²/s), by performing both water- and lipid-soluble metabolome analyses. The findings of this study suggest that manipulating light intensity could be a simple yet effective technique for controlling the production of health-beneficial metabolites in aquaculture microalgae, which can be readily implemented by aquaculture producers.

## Methods

### Culture experiment of chaetoceros gracilis

*Chaetoceros gracilis* strain UTEX LB 2658 was procured from the culture collection of microalgae at the University of Texas at Austin (UTEX, US) and maintained axenically in Daigo’s Artificial Seawater SP (FUJIFILM Wako Pure 81 Chemical Corp., Osaka, Japan) supplemented with Daigo’s IMK medium (FUJIFILM Wako Pure 81 Chemical Corp) at 25 °C under continuous light conditions at 50 µmol photons m^− 2^ s^− 1^ (fluorescent lamps).

For the cultivation of cells for metabolome analysis, 3 mL of late-log phase cells (6.96 × 10^6^ cells/mL) were added to 207 mL of IMK medium (initial cell density 1 × 10^5^ cells/mL). A total of six flasks were prepared, and the cultures were maintained at 25 °C under either normal light (NL, 50 µmol photon/m²/s) or high light (HL, 250 µmol photon/m²/s) conditions; the flasks were agitated at 75 rpm. Fluorescent lamps were used as the light source. The NL and HL conditions were set to approximate the values from previous studies that cultured this species under non-high-light (30–60 µmol photon/m²/s) ^27,28^ and high-light conditions (300 µmol photon/m²/s)^28^, respectively. The three flasks cultured under each light condition were designated as replicate flasks I–III. To investigate shifts in cell density and size during cultivation, 1 mL of culture was sampled on days 1, 2, 3, 4, 7, and 9. The density and size of cells in 40 µL of the sample were measured in bright field cell-counting mode using a Luna FX7 automated cell counter (Logos Biosystems, South Korea). Significant differences in cell density and size on day 9 were analyzed using the Wilcoxon rank-sum test.

### Sample preparation for mass spectrometry

On days 3, 7, and 9 of cultivation, 30, 20, and 20 mL of the culture were sampled, respectively. Cells were collected by centrifugation (25 °C, 16,000 *g*, 10 min), and the supernatant was discarded. An equal volume of artificial seawater was added to wash the cells, and the cells were collected again by centrifugation under the same conditions. The washing step was repeated, and the final supernatant was completely removed. The collected cell pellets were stored at − 80 °C until further analysis.

For the capillary electrophoresis-Fourier transform mass spectrometry (CE-FTMS) analysis of water-soluble compounds, the cell pellets were treated with 1,600 µL of methanol, followed by ultrasonication for 30 s to dissolve the pellet. Next, 1,100 µL of Milli-Q water containing internal standards [H3304-1002; Human Metabolome Technologies (HMT), Tsuruoka, Yamagata, Japan] was added to the cell extract, which was left at room temperature for another 30 s. The mixture was then cooled down on ice and centrifuged at 2,300 ×*g* for 5 min at 4 °C. Subsequently, 700 µL of the supernatant was centrifugally filtered through a 5-kDa cutoff filter (UltrafreeMC-PLHCC, HMT) at 9,100 ×*g*, 4 °C to remove macromolecules. The filtrate was evaporated to dryness under vacuum and reconstituted in 25 µL of Milli-Q water for metabolome analysis at HMT.

Similarly, for the liquid chromatography-Fourier transform mass spectrometry (LC-FTMS) of lipid-soluble compounds, 1,300 µL of ethanol containing internal standards (H3304-1002, HMT) was added to the cell pellets, followed by ultrasonication for 30 s to dissolve the pellet. Afterwards, 1,000 µL of the cell solution was transferred to a fresh tube and resuspended in an equal volume of Milli-Q water. The mixture was further ultrasonicated for 30 s and then centrifuged at 2,300 ×*g*, 4 °C for 5 min. Subsequently, the supernatant was evaporated to dryness under nitrogen and was reconstituted in 400 µL of 50% isopropanol (v/v) for metabolome analysis at HMT.

### Capillary electrophoresis-Fourier transform mass spectrometry analysis

CE-FTMS analysis was conducted according to HMT’s *ω Scan* package, based on the methods described previously^29^. Briefly, this package is a plan to analyze over 1,100 types of water-soluble and ionic metabolites including sugar phosphates, amino acids, nucleic acids, organic acids, vitamins, short- and medium-chain fatty acids, and dipeptides. CE-FTMS analysis in this package was carried out using an Agilent 7100 CE capillary electrophoresis system equipped with a Q Exactive Plus (Thermo Fisher Scientific, Waltham, MA, USA), Agilent 1260 isocratic High-Performance Liquid Chromatography pump, Agilent G1603A CE-MS adapter kit, and Agilent G1607A CE-ESI-MS sprayer kit (Agilent Technologies, Santa Clara, CA, USA). The systems were controlled by Agilent MassHunter Workstation Data Acquisition (Agilent Technologies) and Xcalibur (Thermo Fisher Scientific) and connected by a fused silica capillary (50 μm *i.d.*×80cm total length) with commercial electrophoresis buffer (H3301-1001 and I3302-1023 for cation and anion analyses, respectively, HMT) as the electrolyte. The spectrometer was scanned from *m/z* 60 to 900 in the positive mode and from *m/z* 70 to 1,050 in the negative mode^29^. Peaks were extracted using MasterHands (Keio University, Tsuruoka, Yamagata, Japan), which is an automatic integration software, to obtain peak information, including *m/z*, peak area, and migration time (MT)^30^. Signal peaks corresponding to isotopomers, adduct ions, and other product ions of known metabolites were excluded, and the remaining peaks were annotated according to HMT’s metabolite database based on their *m*/*z* values and MTs. Afterwards, the areas of the annotated peaks were normalized to the internal standards and sample volume to determine the relative levels of each metabolite. The 83 compounds listed in Table S1 were absolutely quantified based on one-point calibrations using their respective standard compounds, as they generally induce health-beneficial effects.

### Liquid chromatography-Fourier transform mass spectrometry analysis

LC-FTMS analysis was conducted according to HMT’s *LC-ω Scan* package, based on the methods described previously^31,32^. Briefly, this package is a plan to analyze over 450 types of lipid-soluble and neutral metabolites including fatty acids, acylcarnitines, bile acids, and steroid derivatives. LC-FTMS analysis in this package was performed using the Vanquish Flex Ultra-High-Performance Liquid Chromatography System (Thermo Fisher Scientific). The systems were controlled by Xcalibur (Thermo Fisher Scientific) and connected by an ODS column (2 mm i.d. × 50 mm, 2 μm). The spectrometer was scanned from m/z 100 to 1,500, and peaks were extracted using MasterHands (Keio University, Tsuruoka, Yamagata, Japan) to obtain peak information including m/z, peak area, and retention time (RT)^30^. Signal peaks corresponding to isotopomers, adduct ions, and other product ions of known metabolites were excluded, and the remaining peaks were annotated according to HMT’s metabolite database based on their m/z values and RTs. Subsequently, the areas of the annotated peaks were normalized to the internal standards and sample amount to determine the relative levels of each metabolite.

### Comparison of compound composition and statistical analyses

Welch’s t-test was used to statistically compare the relative abundance of metabolites between samples. Hierarchical cluster analysis and principal component analysis (PCA) were performed by HMT’s proprietary MATLAB and R programs, respectively^33^. Briefly, hierarchical clustering was performed using the unweighted pair group method with arithmetic mean (UPGMA), where the inter-cluster distance was calculated as the average of all pairwise distances between metabolites. Prior to analysis, all metabolite values were standardized (mean = 0, SD = 1), and non-detectable (N.D.) values were replaced with a minimal value (2⁻⁵²). Principal component analysis (PCA) was performed using a MATLAB-based program, where metabolite data were mean-centered and scaled before computing eigenvectors from the correlation matrix; the ‘princomp’ function was used as part of this program. Detected metabolites were mapped on metabolic pathway using VANTED version 2.1.0 (https://cls.uni-konstanz.de/software/vanted/)^34^.

## Results

### Shifts in cell densities and sizes under high and normal light conditions

**Fig. 1:**
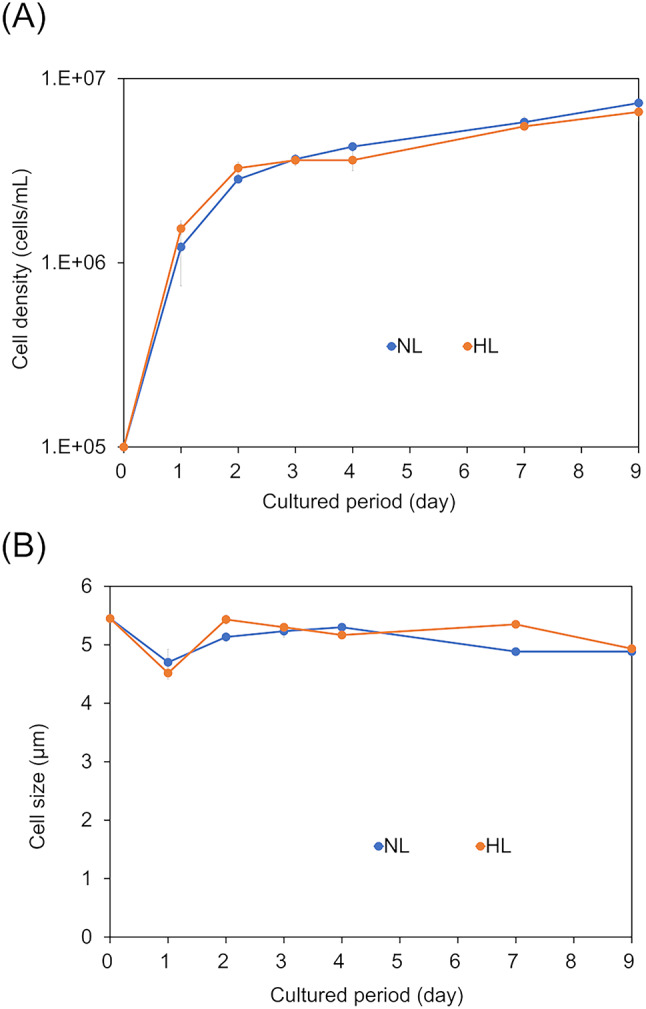
Shifts in cell density and size during the culture period. (**A**) Shifts in cell density (cells/mL). The horizontal axis shows the cultured period (day), and the vertical axis represents the cell densities. Cell density was measured using a LUNA-FX7 cell counter. The average density from triplicate flasks are shown for each day. Error bars indicate the standard deviation in cell density (cells/mL). (**B**) Shifts in cell size (µm). Cell size was measured using a LUNA-FX7 cell counter. The average size from triplicate flasks are shown for each day. Error bars indicate the standard deviation in cell size (µm).

Firstly, to investigate the effects of differences in light intensity on the cell yield of *C. gracilis*, we examined the changes in cell density and size under high light (HL) and normal light (NL) conditions. The cell density under HL conditions reached the late exponential growth phase (6.60 ± 0.71 × 10^6^ cells/mL) by day 9. A similar trend was observed under NL conditions (7.37 ± 0.58 × 10^6^ cells/mL on day 9), and there was no significant difference in cell density on day 9 (*p* = 0.4) (Fig. 1a). As *C. gracilis* is generally harvested during the late exponential growth phase for aquaculture feed^35^, the ninth day of cultivation is defined as the harvest period. In terms of cell size, it decreased from day 0 to day 1 under both HL (4.51 ± 0.10 μm) and NL (4.7 ± 0.21 μm) conditions. However, after day 1, it gradually stabilized between 4.93 ± 0.06 to 5.43 ± 0.08 μm under HL conditions and between 4.88 ± 0.06 to 5.30 ± 0.05 μm under NL conditions, with no significant difference in cell size observed on day 9 between the light conditions (*p* = 0.3017) (Fig. 1b). These results revealed that NL and HL conditions did not significantly affect cell density and size, suggesting that the light conditions set in this study did not cause clear photoinhibition and that there is no substantial difference in cell yield under these two conditions.

### Changes in the composition of low-molecular-weight compounds under high and normal light conditions

To reveal the effect of light intensity differences on the production of water-soluble and lipid-soluble LMW compounds in *C. gracilis*, we performed CE-FTMS (for water-soluble compounds) and LC-FTMS (lipid-soluble compounds) analyses. CE-FTMS analysis facilitated the detection of a total of 279 and 282 water-soluble LMW compounds in the NL and HL samples, respectively, on any of days 3, 7, and 9 (Table S1). On the other hand, LC-FTM analysis enabled the detection of 84 and 77 lipid-soluble LMW compounds in the NL and HL samples (Table S2), respectively. The relative abundance of these compounds is presented in Supplementary Tables 1 and 2.

**Fig. 2:**
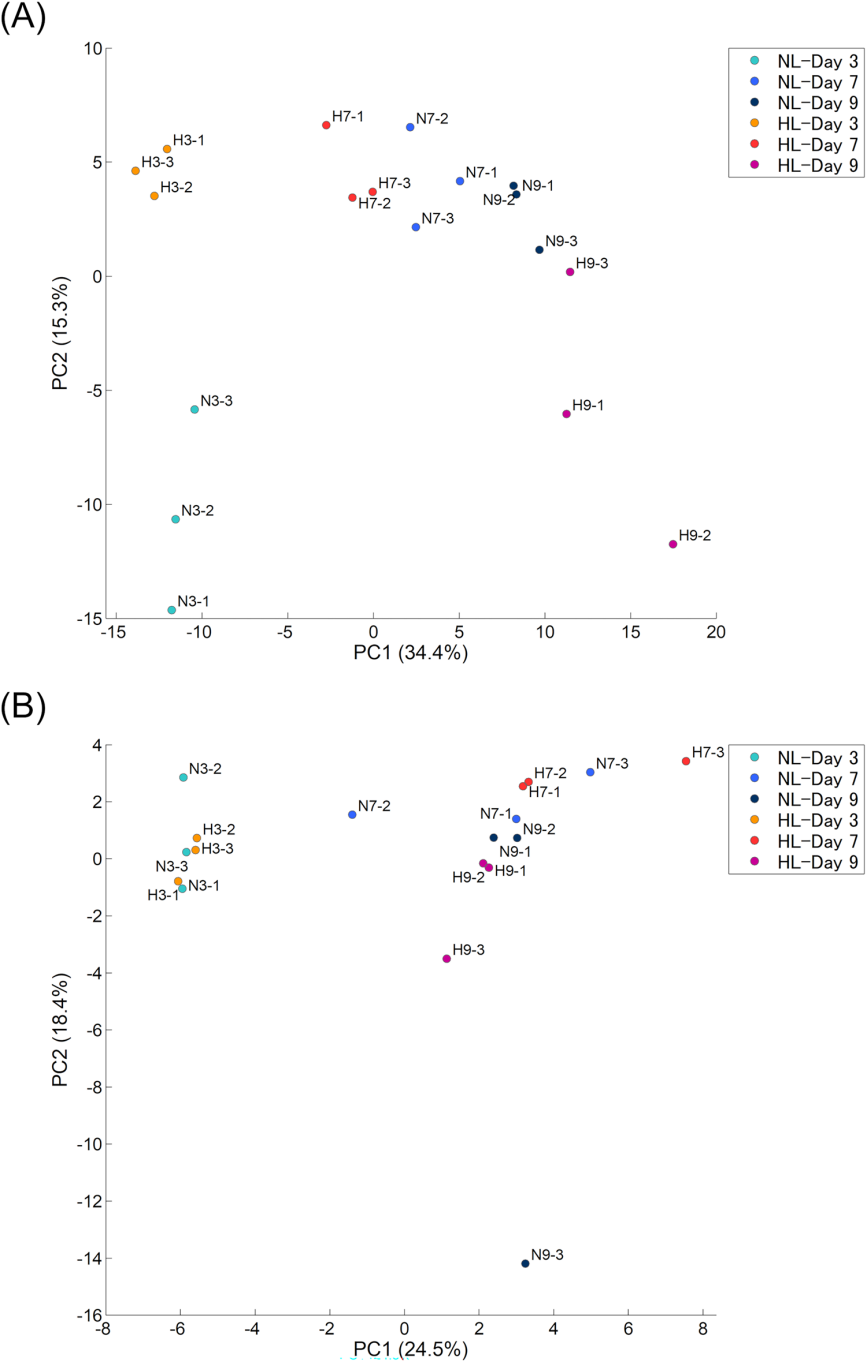
Comparison of compound compositions in each sample. Water-soluble compounds (**a**) and lipid-soluble compounds (**b**). The area of detected peaks (compounds) in each sample was normalized, and principal component analysis was performed. Samples are distinguished by colors based on the treatments and culture periods. The sample ID indicates sample condition (N for NL and H for HL), cultured period (3, 7, or 9), and the number of replicate flasks.

The results of PCA analysis, which was conducted to determine the effects of varying light intensities on the overall composition of LMW compounds, are presented in Fig. 2. Regarding the water-soluble compounds, PC1 (34.4%) and PC2 (15.3%) tended to be associated with culture duration and light intensity, respectively. Compounds with high factor loadings on PC1 were ɤ-Glu-Val, ɤ -Glu-Phe, N6,N6,N6-trimethyllysine, ɤ -Glu-Ile/ɤ -Glu-Leu and cytidine diphosphate, while those with high factor loadings on PC2 were N-acetyltyrosine, p-hydroxyphenylacetylglycine, and 2-oxoglutaric acid (Table S3). The latter three compounds are candidates for the light intensity-dependent accumulation, which is the main focus of this study. Focusing on the HL samples, the replicates on the same day samples were closely plotted (Fig. 2a), suggesting the compound composition changed tightly linking to the duration of the culture period. However, while the NL samples were also clustered and plotted according to each replicate, the plots from days 7 and 9 were grouped together, indicating that there may have been fewer changes in compound composition compared to those in the HL samples. When comparing the light conditions, the plots were already separated on day 3; moreover, the NL and HL samples were distinctly plotted on the harvest day (day 9), suggesting that varying light intensities affected compound composition.

In the case of lipid-soluble compounds, PC1 (24.5%) also tended to be associated with culture duration, while PC2 (18.4%) was linked to light intensity. However, upon examining the plot positions of each sample, they displayed different behavior compared to the water-soluble compounds (Fig. 2b). On day 3, all of the HL and NL samples were clustered and plotted together. Although the HL and NL plots shifted thereafter, the replicates of these samples were not clustered, resulting in less clear differences between the light conditions compared with those observed in the water-soluble compounds. Therefore, although compounds 1,2-dipalmitoyl-glycero-3-phosphoglycerol-3, α-tocopherol, and sphinganine have high factor loadings on PC1, and tetradecanedioic acid, decanoic acid, and 2-arachidonoylglycerol-2 have high factor loadings on PC2 (Table S4), it remains uncertain whether these compounds are truly influenced by culture duration or light intensity.

**Fig. 3:**
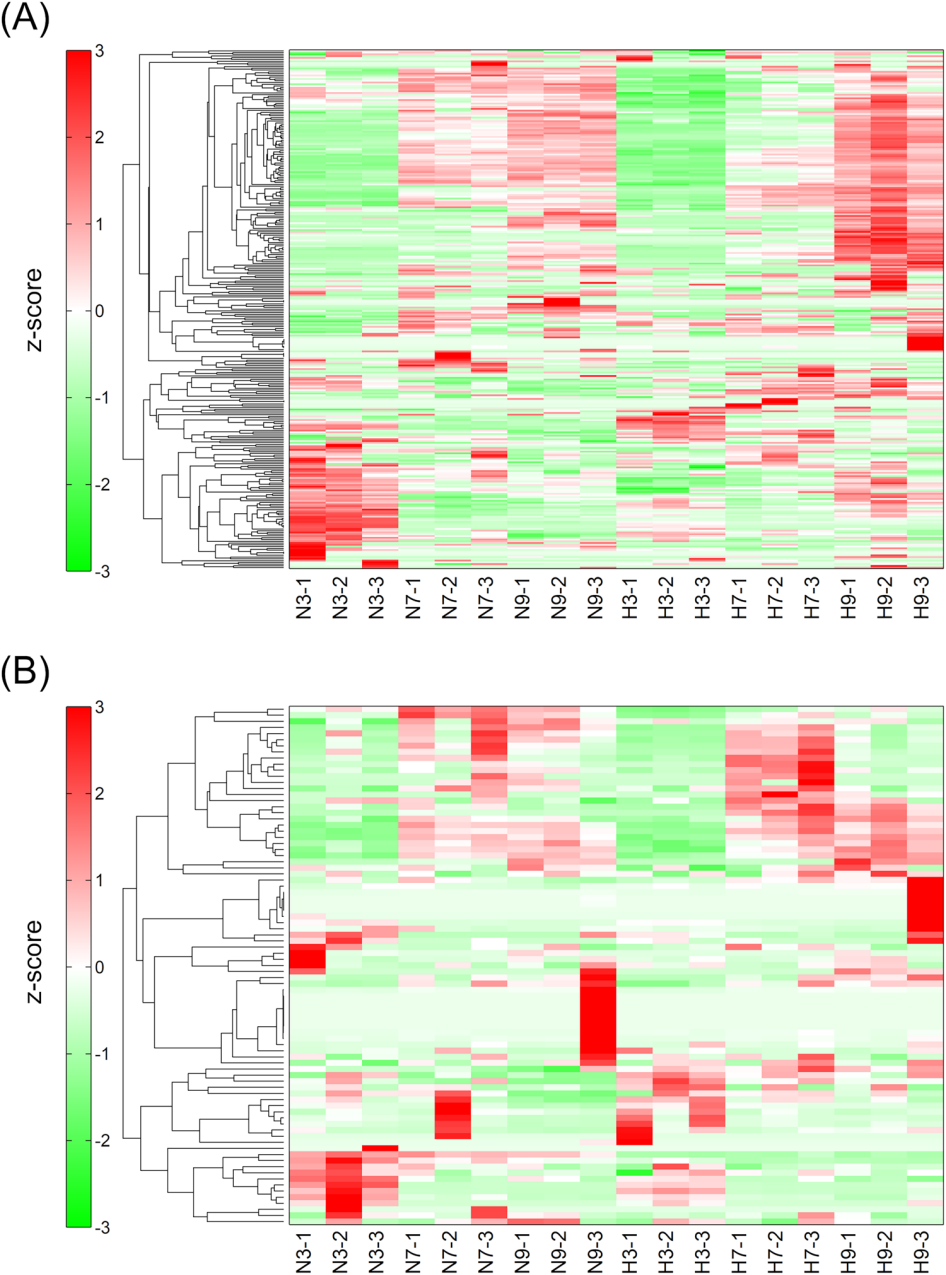
Distribution of compounds across samples. Water-soluble compounds (**a**) and lipid-soluble compounds (**b**). The horizontal axis shows samples, and the vertical axis represents the detected peaks (compounds). For hierarchical clustering analysis, the area of detected peaks (compounds) in each sample was normalized, and the distances of normalized peak areas were calculated. The dendrogram represents the distance of each peak. The colors in the figure represent z-scores, which were calculated with normalized peak area. The darker green indicates smaller values, and the darker red indicates larger values. The sample ID indicates sample condition (N for NL and H for HL), cultured period (3, 7, or 9), and the number of replicate flasks.

To determine whether these changes in compound composition are due to variations in the abundance of specific compounds, changes in relative abundance of each compound were analyzed at the individual compound level (Fig. 3). For water-soluble compounds, there was minimal variation between replicates, and the accumulated compounds tended to change with the culture period (Fig. 3a). Even on the same cultivation day, the accumulated compounds differed between the HL and NL conditions, consistent with the results of the PCA analysis. Thus, specific compounds were considered to accumulate according to the culture period and the light intensity. On the contrary, for lipid-soluble compounds, there was variation between replicates (Fig. 3b), and a less clear difference in the compound composition between NL and HL samples was observed, supporting the findings of the PCA analysis. It is possible that the composition of lipid-soluble compounds may be less sensitive to light intensity compared to water-soluble compounds. However, the overall trend was similar to that observed for water-soluble compounds, with specific compounds accumulating based on the cultured period and the light intensities.

### Low-molecular-weight compounds accumulated on the harvest day

**Fig. 4:**
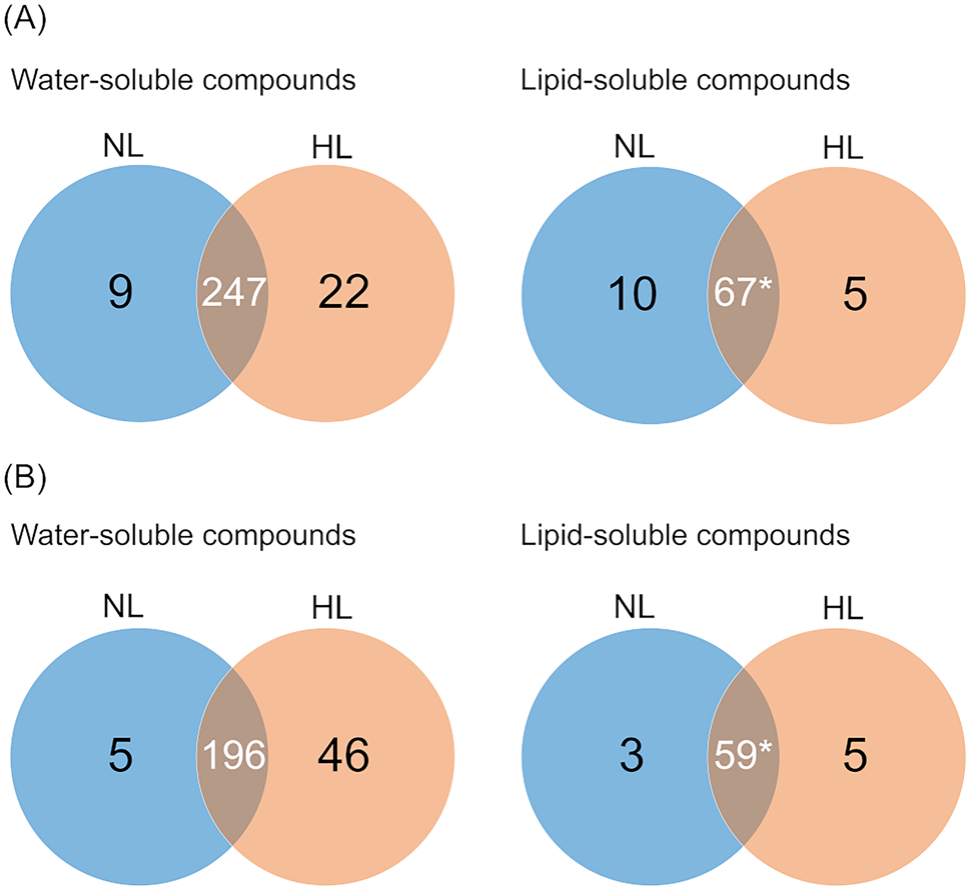
Venn diagram comparing the number of compounds detected under each light intensity in the samples on day 9. (**a**) Presence and absence of compounds based on relative abundance. (**b**) Compounds detected in both normal light (NL) and high light (HL) conditions, but showed significantly higher relative abundance in any sample (*p* < 0.05). *: The number of compounds that did not show significantly different relative abundance between NL and HL. The water- and lipid-soluble compounds are shown separately.

Given the differences in the relative abundance of each compound throughout the culture period and between the light conditions, the compounds that were detected on day 9 were focused on to identify the compounds that accumulated at the harvest period. Of the 247 water-soluble compounds detected under both NL and HL conditions, 22 and 9 compounds were detected under HL and NL conditions, respectively (Fig. 4a, left). Moreover, of the 247 commonly detected compounds, 46 had significantly higher relative abundance under HL conditions, while only 5 did under NL conditions (Fig. 4b, left). Meanwhile, of the 67 lipid-soluble compounds that were detected under both NL and HL conditions, 5 were detected only under HL conditions, while 10 were only detected under NL conditions (Fig. 4a, right). Among the 67 commonly detected compounds, 5 and 3 compounds had significantly higher relative abundance under HL and NL conditions, respectively (Fig. 4b, right). These compounds that either appeared exclusively or had significantly higher relative abundance in the day 9 samples under either HL or NL conditions were labeled as HL- (78 in total) or NL-specific compounds (27 in total) (Supplementary Tables 5 and 6). Among these, 8 HL- and 9 NL-specific compounds were detected only in the day 9 samples (Supplementary Tables 1 and 2). The accumulation of these compounds may be strongly influenced not only by light intensity but also by the duration of cultivation. Note that the candidates for HL-related compounds refined through PCA, such as N-acetyltyrosine, p-hydroxyphenylacetylglycine, and 2-oxoglutaric acid, were not included among the HL-specific compounds.

In contrast, 196 and 59 of the water- and lipid-soluble compounds demonstrated no significant difference in relative abundance between HL and NL conditions (Supplementary Tables 1 and 2). These were referred to as non-specific compounds. The water-soluble non-specific compounds included four essential amino acids (His, Lys, Met, and Phe), and the lipid-soluble non-specific compounds included two essential fatty acids (arachidic acid and linoleic acid). These compounds may have been produced independently of the differences in light intensity set in this study.

### Low-molecular-weight compounds specifically accumulated under high or normal light conditions

As the HL- and NL-specific compounds were considered to be specifically produced under each light condition at the harvest period, previous studies and public databases were referred to in identifying the compounds with health benefits to humans and aquatic organisms. It was found that 14 out of the 78 HL-specific compounds and 9 out of the 27 NL-specific compounds have health benefits (Tables 1 and 2).

**Table 1:** List of high light (HL)-specific compounds identified as health-beneficial metabolites. The table includes the effects of each compound, a comparison of relative quantities in the samples on day 9, and their statistical significance. In the metabolome analysis, two peaks were annotated for piperine, and since it was unclear which was the isomer, they were named piperine-1 and piperine-2.

Compounds	Health-beneficial effect	Relative abundance (peak area ratio to the internal standard)		
		NL	HL	*p*-value
Citric acid	Antioxidant effect	3.878	6.186	0.00962
Citrulline	Muscle and metabolic health in susceptible/elder populations	0.136	0.182	0.0471
N5-Ethylglutamine (Theanine)	Reduction of stress, antioxidant	0.0357	0.0553	0.0321
Retinoic acid	Promoting recovery from photodamage to the skin	0.00392	0.0110	0.0373
Carnosine	Antioxidant and antiaging effect	N.D.	0.0141	-
Creatine	Enhancing exercise performance	N.D.	0.0914	-
GABA	Inhibitory neurotransmitter	N.D.	0.0672	-
Ile	Essential amino acid for humans	8.653	11.939	0.0411
Leu	Essential amino acid for humans	10.230	15.112	0.0145
Thr	Essential amino acid for humans	4.474	5.632	0.0478
Trp	Essential amino acid for humans	0.660	0.929	0.0179
Val	Essential amino acid for humans	11.913	21.437	0.0128
β-Ala	Improvement of exercise performance	0.179	0.392	0.0197
Piperine-2	Antioxidant and anti-inflammatory effect	N.D.	0.270	-

**Table 2:** List of normal light (NL)-specific compounds identified as health-beneficial metabolites s. The table includes the effects of each compound, a comparison of relative quantities in the samples on day 9, and their statistical significance.

Compounds	Health-beneficial effect	Relative abundance (peak area ratio to the internal standard)		
		NL	HL	*p*-value
Pyroglutamine	Risk-reducing effect for COVID-19	0.00460	N.D.	-
Ribavirin	Antiviral agent	0.00240	N.D.	-
6-Keto-prostaglandin E1	Antithrombotic effects	0.184	0.0869	0.00235
Fucoxanthin	Antioxidant and anti-inflammatory effect	2027.026	1024.736	0.00711
Palmitoleic acid	Anti-inflammatory properties in human endothelial cells	0.0751	0.0300	0.0377
Cholestenone	Antibiotic against Helicobacter pylori	0.636	N.D.	-
Linolenic acid	Essential fatty acid for humans	0.000429	N.D.	-
Nobiletin	Anti-inflammatory effects	0.00633	N.D.	-
Prostaglandin D2	Anti-inflammatory effects	0.0389	N.D.	-

Ile, Leu, Thr, Trp, and Val are the essential amino acids^36^ detected in the HL-specific compounds. Citric acid and carnosine are known for their antioxidant and antiaging effects^37,38^. Citrulline is beneficial to muscle and metabolic health in susceptible and elderly populations^39^. N5-Ethyl-glutamine (theanine) is a stress-reducing compound^40^. Retinoic acid is considered effective in promoting the recovery of the skin from photodamage^41^. Supplementation with creatine plays a role in enhancing exercise performance^42^. Gamma-aminobutyric acid (GABA) is an inhibitory neurotransmitter that contributes to the prevention and improvement of vascular diseases^43^. Piperine possesses antioxidant and anti-inflammatory properties^44^. Among these aforementioned compounds, carnosine, GABA, and piperine were only detected in the HL samples on day 9 (Table S5).

On the other hand, the NL-specific compounds that have health benefits include the following: pyroglutamine, which is known to have a risk-reducing effect for COVID-19^45^; ribavirin, an antiviral agent effective at treating hepatitis C^46^; 6-keto-prostaglandin E1, whose antithrombotic properties prevent platelet clot formation^47^; fucoxanthin, which is widely known for its antioxidant and anti-inflammatory properties^48^; palmitoleic acid, with anti-inflammatory properties in human endothelial cells^49^; cholestenone, which acts as an antibiotic against *Helicobacter pylori*^50^; linolenic acid, an essential fatty acid for humans^51^; nobiletin, which has anti-inflammatory and anti-tumor effects and is beneficial for treating Alzheimer’s and Parkinson’s diseases^52–54^; and prostaglandin D2, which exhibits potential as an anti-inflammatory agent^55^. Among these compounds, cholestenone was detected only in the NL samples on day 9 (Table S5).

**Fig. 5:**
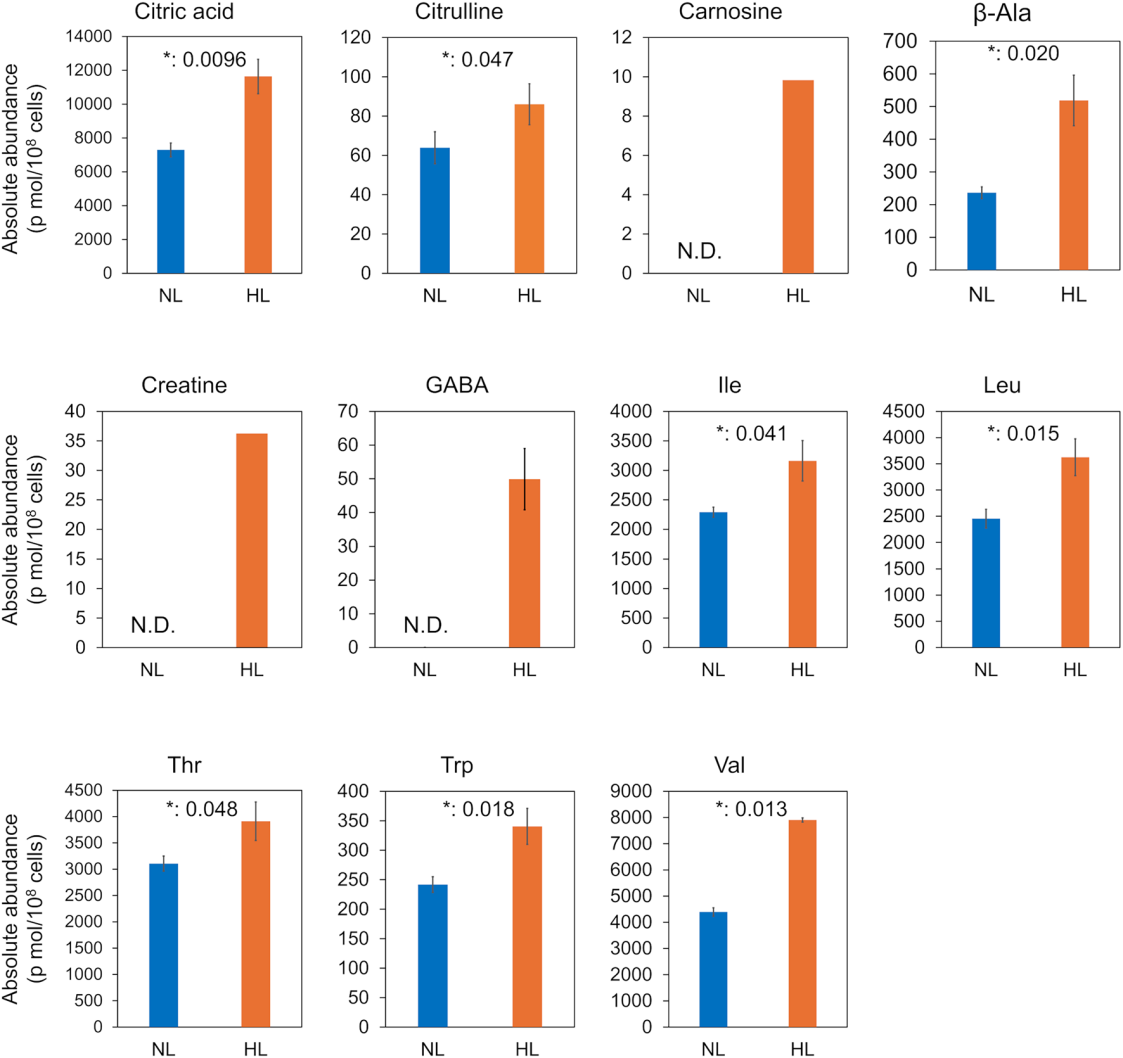
Absolute quantification of water-soluble substances in the samples on day 9. For citric acid, citrulline, carnosine, β-Ala, creatine, GABA, Ile, Leu, Thr, Trp, and Val, internal standards with known concentrations were analyzed together using capillary electrophoresis-Fourier transform mass spectrometry. The absolute amounts in the samples were calculated by referencing the peak areas of the internal standards using single-point calibration. N.D: Not detected. *p*-values are indicated by asterisks.

Since the HL-specific compounds citric acid, citrulline, carnosine, β-Ala, creatine, GABA, Ile, Leu, Thr, Trp, and Val are considered highly beneficial for both humans and aquatic organisms, their absolute abundance on day 9 was quantitatively compared (Fig. 5). All of these compounds exhibited significantly higher absolute quantities in the HL samples than in the NL samples (*p* < 0.05) or were even only detected in the HL samples.

## Discussion

In the present study, comprehensive metabolomic analysis was performed to determine the effect of the difference in the light intensities during cultivation on the type and quantity of health-beneficial metabolites produced by *C. gracilis*, an important microalgal species for aquaculture feed. The results revealed that although the difference in the light intensities did not significantly affect the overall feed yield, several compounds, including health-beneficial ones, accumulated in a light-intensity-specific manner. Additionally, the absolute abundance of citric acid, citrulline, carnosine, β-Ala, creatine, GABA, Ile, Leu, Thr, Trp, and Val were significantly higher under HL conditions than under NL conditions, emphasizing the potential different nutritional characteristics of *C. gracilis* when cultured under different light intensities. Although it remains unclear whether the concentrations of these metabolites are sufficient as aquaculture feed, a more detailed evaluation could be achieved in the future by conducting actual fish and shellfish production using these microalgae. Additionally, as absolute quantification was not performed for some of the health-beneficial metabolites detected in this study, their exact accumulation levels remain unknown. Nevertheless, as significant differences in relative quantities were observed, it cannot be ruled out that microalgal feed with different nutritional characteristics can still be produced, and their effects need to be evaluated through practical fish and shellfish production. Interestingly, there were health-beneficial metabolites not only known to be produced by microalgae responding to changes in light intensity but also those scarcely reported to be present in microalgal cells.

Ten of the following health-beneficial metabolites identified in this study have been reported to be produced by microalgal cells in response to light intensity. Fucoxanthin is the most notable example of a microalgae-derived compound related to light intensity. Fucoxanthin is a characteristic pigment in diatoms and has antioxidant properties^56^. In this study, the relative abundance of fucoxanthin was higher under NL conditions than under HL conditions, suggesting that degradation and/or a decrease in production of fucoxanthin occurred at 250 µmol/m²/s. This result is consistent with the common notion that a light intensity of 10–100 µmol/m²/s is optimal for several diatom species^56^. As GABA is known to be produced by microalgae, such as red algae, and has been reported to alleviate severe light stress within microalgal cells^57,58^, it likely contributed to the reduction of oxidative stress under HL conditions. The relationship between light intensity and the production of linolenic acid and palmitoleic acid has been described in previous studies^15,59–61^. In this study, the production of linolenic and palmitoleic acids decreased under HL conditions, consistent with the results of previous studies involving the green microalga *Chlorella protothecoides* (linoleic acid) and the diatom *Staurosira* sp. (palmitoleic acid)^59,60^. On the other hand, there have been studies reporting that the accumulation of linoleic acid in *Euglena gracilis* and palmitoleic acid in the ochrophyte *Nannochloropsis salina* increased with higher light intensity^15,61^. However, the “high light” defined as 610 footcandles (approx. 6,595 lx) in the former, when converted assuming white fluorescent lighting, equates to 87–92 µmol photons/m²/s^62^, which nearly corresponds to the NL condition in our study. In the latter case, while high light intensity of 250 µmol photons/m²/s was indeed optimal during the exponential phase, when comparing the production yield during the late linear growth phase corresponding to the harvest period in this study, the peak occurred at 25–50 µmol photons/m²/s. These values also match the NL condition of our study. This suggests that, despite some variations across species and experimental conditions, these fatty acids are likely to accumulate at the NL condition (50 µmol photons/m²/s). Retinoic acid, an antioxidant in plant cells^63^, is known to increase during natural microalgal blooms, suggesting that it may also increase in microalgal cells under certain conditions^64^. The fact that the occurrence of blooms responds to certain light intensities explains the abundance of retinoic acid under HL conditions. The origin of Ile, Leu, Thr, Trp, and Val is likely associated with amino acid recycling; however, pinpointing the exact cause remains challenging. Considering that fucoxanthin decreased under HL conditions, indicating potential light inhibition, it could be hypothesized that these amino acids are derived from the degradation of fucoxanthin or chlorophyll/light-harvesting complexes.

Furthermore, there were health-beneficial metabolites known to be produced by microalgae; however, their relationship with light intensity is not clarified. Citrulline is known to be produced by *Chlorella vulgaris* and *Chlorella sorokiniana*^65,66^. Although its intracellular function in microalgae is not well understood, it acts as a strong antioxidant in melons^67^, suggesting it may perform a similar role in microalgal species. Citric acid plays a role in the tricarboxylic acid cycle and other metabolic pathways. It is also known to mitigate oxidative stress^68^, likely contributing to the reduction of oxidative stress under HL conditions. Cholestenone has been reported to be produced by red algae^69^; however, the mechanisms by which light intensity affects its production remain unclear. In terrestrial plants, changes in the photoperiod during cultivation affect the production of cholestenone^70^, indicating a possible relationship with light. Pyroglutamine is known to be produced in algal cells, such as the brown seaweed *Ishige okamurae* and the green microalgal model species *Chlamydomonas reinhardtii*^71,72^; however, its relationship with light intensity is unknown. Although prostaglandin D2 is also produced in algal cells^73^ and is involved in multiple physiological processes in animal cells, its role and relationship with light intensity in algal cells remain unclear^73^.

Interestingly, some health-beneficial metabolites that were not commonly reported to be produced by microalgae were detected in this study. So far, carnosine, a histidine-containing dipeptide, has been exclusively found in animals^74^. However, a previous study reported that exogenous application of carnosine alleviated drought stress in a terrestrial plant, Bermudagrass, suggesting its antioxidant properties in plant cells^75^. In this study, β-alanine (HL-specific) and histidine, precursors of carnosine^76^, have been detected, suggesting the potential presence of the metabolic pathway. Although the relationship between these compounds and light intensity is not well known, it is possible that their biosynthetic pathways could be regulated by light intensity. The production of theanine within microalgal cells is rarely reported; nonetheless, its antioxidant properties in tea leaves^77^ could potentially alleviate oxidative stress under HL conditions. However, theanine concentrations tend to increase under shaded or low light conditions in tea leaves^77–79^, indicating that the light-responsive production mechanism of theanine may differ between tea leaves and microalgae. Similar to the detection of carnosine, glutamine, a precursor of theanine, has been detected, although it is not HL-specific, suggesting that the metabolic pathway may exist. Similarly, the production of piperine and nobiletin by algal cells has not been reported. These compounds are antioxidants derived from plant cells^44,80^ and might have played a role in mitigating oxidative stress under HL conditions in this study. For piperine, its precursors piperidine and lysine^81^ have been detected (not HL-specific compounds), suggesting that its metabolic pathway may exist. Regarding nobiletin, related compounds, such as 3’,4’,6-demethyl nobiletin and 7- demethyl nobiletin^82^ were not detected in this study (Table S1&S2). While the detection of nobiletin cannot rule out the risk of contamination, the related compounds were not listed in the database used for this study and may not have been annotated even if they were present. Ribavirin, an antiviral agent synthesized in 1972^83,84^, is unlikely to be produced by algal cells. According to previous studies, this compound has been released into the environment and has accumulated in certain aquatic environments^85^. On the other hand, since the culture medium used in this study was consistently based on purified water, it cannot be ruled out that the compounds may have been contaminated during the analysis rather than accumulated during culture. Although this species may possess the novel metabolic pathway to synthesize ribavirin, these possibilities remain issues that should be investigated further in future studies. Additionally, the low levels of ribavirin observed under HL conditions are consistent with its reported photodegradability in previous studies^85^. The production of 6-keto-prostaglandin E1 in either microalgal or plant cells has not been reported so far; thus, further studies are required to elucidate the role of 6-keto-prostaglandin E1 in this species. Similar to the case of nobiletin, the precursor compound, prostacyclin^47^, was not detected and not listed in the database used for this study (Table S1&S2). Thus, there are two possibilities: contamination may occurred, and its precursor prostacyclin may have been present but remained unannotated.

It is noteworthy that these compounds possess diverse health-beneficial functions. For instance, the HL-specific compounds exhibit antioxidative, skin-regenerating, anti-aging, anti-inflammatory, athletic-performance-improving, and muscle-enhancing properties (Table 1). Meanwhile, the NL-specific compounds not only exhibited antioxidative and anti-inflammatory properties but were also effective against COVID-19, hepatitis C virus, and *Helicobacter pylori* (Table 2). These results indicate that there are advantages associated with each cultivation light intensity. Therefore, aquaculture producers could adjust light intensity according to their goals. Future studies should be conducted to investigate the extent to which these compounds accumulate in seafood. Furthermore, it has been reported that the oral administration of *C*,* gracilis* alleviated liver lipid accumulation in rats fed with a high-sucrose and cholesterol diet. If this species were to be utilized not only as aquaculture feed but also as a food resource, the aforementioned health benefits and medicinal effects may offer potential additional value.

Indeed, this study reveals the presence of microalgae-derived health-beneficial metabolites that accumulate in response to light intensity, which has rarely been reported before. However, there are several challenges. The study primarily focuses on metabolome analysis, which elucidates the outcomes of the synthesis and degradation of various compounds. Consequently, the mechanisms underlying the accumulation of health-beneficial metabolites in response to light intensity remain unclear. This issue may be addressed by combining data from transcriptomics, proteomics, and flux analysis using^13^C stable isotopes, which represents a future research direction. Furthermore, if the goal is to mass-produce and commercialize specific compounds described above, genetic engineering methods such as gene modification would be preferable. For example, in the production of carnosine, the use of recombinant *Escherichia coli* has successfully achieved a production level of 133.2 mM in a 5 L bioreactor^86^. On the other hand, there is a possibility that the results of this study could be highly compatible with those methods for the following reasons. In this study, it was revealed that the production levels of numerous compounds vary with differences in light intensity. Therefore, as mentioned above, by applying transcriptomics, proteomics, and^13^C stable isotope analysis in addition to metabolomics to microalgae cultured under varying light conditions, it may be possible to identify the regulatory factors controlling these production levels, thereby providing valuable information for genetic engineering methods.

However, as the data from this study also indicate, light intensity affects several metabolic processes, making the identification of specific regulatory factors challenging. Hence, the ability to control the quantity of health-beneficial metabolites through light intensity alone, as demonstrated in this study, is advantageous. Furthermore, this method holds several advantages over genetic engineering methods for commercial applications: (1) unlike recombinant organisms, there are no regulatory restrictions; (2) it does not require breeding using specialized techniques; (3) the quantities of multiple compounds can be enhanced; and (4) there is no significant difference in growth quantity but allowing for the modification of chemical composition. Altogether, this approach potentially enables adjustments to the added value of the feed without significantly altering the cell yield. This study did not calculate the costs associated with cultivation, and thus whether aquaculture producers can feasibly implement the methods has not been thoroughly examined. However, the light intensity set in this study is feasible for several cultivation equipment used in practical algae production, such as raceway ponds and photobioreactors^87^, suggesting that the cost for electricity may not be a major issue. Therefore, this approach may be implemented even by aquaculture producers who may not possess advanced technical expertise or specialized equipment. Furthermore, as noted above, it cannot be ruled out that the concentrations of focal health-beneficial metabolites in actual aquaculture production may be too low to exert health-promoting effects on fish or humans. However, it is also known that variations in the amounts of nutrients such as silicon, nitrogen, and phosphorus, as well as cultivation temperature, can affect the accumulation of lipids and other compounds^88,89^ By controlling these cultivation conditions in addition to light intensity, there is potential to create a synergistic effect that more efficiently increases the production of health-beneficial metabolites.

Although adjusting light intensity is a classical technique, combining it with state-of-the-art metabolome analysis, as demonstrated in this study, could lead to the discovery of new added value for aquaculture feed. In the future, it will be important not only to expand the evaluation of feed value, including macronutrients, under a broader range of culture conditions, such as varying light intensities, but also to verify, through practical fish and shellfish production, whether these microalgal feeds actually influence the health of fish and humans.

## Supplementary Information

Below is the link to the electronic supplementary material.

Supplementary Material 1

## Data Availability

The raw data supporting the conclusions of this article will be made available from the corresponding author on reasonable request.
